# Clinical applications of concentrated growth factors membrane for sealing the socket in alveolar ridge preservation: a randomized controlled trial

**DOI:** 10.1186/s40729-022-00448-w

**Published:** 2022-11-01

**Authors:** Yumeng Liu, Xiaoming Li, Changwei Jiang, Huiying Guo, Guisheng Luo, Yangyang Huang, Changyong Yuan

**Affiliations:** 1 School of Stomatology, Xuzhou Medical University, Xuzhou, 221004 Jiangsu China; 2 Department of Oral Implantology, The Affiliated Stomatological Hospital of Xuzhou Medical University, 130 Huaihai West Road, Xuzhou, 221000 Jiangsu China

**Keywords:** Dental implant, Concentration growth factors, Alveolar ridge preservation, Soft tissue healing

## Abstract

The purpose of this study was to evaluate the efficacy of concentrated growth factor (CGF) membrane for the sealing of alveolar socket in alveolar ridge preservation (ARP). A total of 22 patients with 24 alveolar sockets were recruited and divided randomly into CGF group and Bio-Gide collagen membrane group. The soft tissue wound healing rate was calculated using intraoral scanner at 3, 7, and 14 days after ARP, and the bone resorption volume at 1, 3, and 5 mm below the alveolar ridge was measured by CBCT at 6 months postoperation. The keratinized gingival width was also measured before and 6 months after ridge preservation. In terms of soft tissue healing rate, the CGF group exhibited significant higher than that of Bio-Gide group at both 7 and 14 days after surgery (*P* < 0.05). However, there was no significantly different in bone resorption rate and the width of keratinized gingival after 6 months (*P* > 0.05). Therefore, the use of CGFs membranes for wound closure in ARP is a reliable method, but more clinical data are needed to prove it.

## Introduction

Alveolar ridge resorption is regarded as the common phenomenon after teeth extraction. It was reported that the buccal bone plate is less than 1 mm in thickness in most sites in the anterior maxilla. In addition, nearly half of the tested patients had a labial plate thickness of only 0.5 mm [1]. Extensive resorption of the alveolar ridge occurs in the first 3 months after tooth extraction [2]. Literatures showed that vertical dimension decreased by 11–22% at 3 months, and horizontal dimension decreased by 32% at 3 months and 29–63% at 6–7 months [3]. Such bone resorption may cause the staged guided bone regeneration (GBR), which increases the surgical difficulty and potentially results in the occurrence of a hematoma and postoperative pain [4].

Alveolar ridge preservation (ARP) is considered as an effective method to reduce bone resorption and maintain alveolar bone morphology post-extraction, which includes the socket filling with different biomaterials [5] and sealing with closure materials to prevent the early loss of the underlying biomaterial [6]. Tight suturing of the wound will not only decrease the risk of wound infection, but also prevent early shedding of biomaterials, which possibly affect subsequent bone tissue contours.

Three sealing materials are frequently used in ARP: autogenous tissue, absorbable and non-resorbable collagen membranes [7]. Studies showed that there is no significant difference among them in ARP, but each has drawbacks. Autogenous tissue is mainly derived from patient's palatal soft tissue which causes another surgical damage, postoperative bone exposure, and additional pain [8]. The non-resorbable e-PTFE barrier (e-PTFE, Gore-Tex^®^) membranes often give rise to soft tissue dehiscence and need to be removed in time, which increase the risk of infection [9]. Resorbable collagen membranes are user-friendly, and have the advantage to increase keratinized tissue thickness. Nevertheless, animal origin and high price limit their application in ARP [10].

Growth factor (GF) is reserved in the alpha granules in platelet, which contains high quantities of GFs, such as vascular endothelial growth factor (VEGF), transforming growth factor-β1 (TGF-β1) and β2 (TGF-β2), fibroblast growth factor (FGF), platelet-derived growth factor (PDGF) and insulin-like growth factor (IGF) [11, 12]. GFs play an important role in the modulation of healing after dental implant placement, include stimulation of cell proliferation, matrix remodeling and angiogenesis [13, 14].

Concentration growth factors (CGF) was developed by Sacco in 2006, and is derived from the centrifuged peripheral venous blood, contains blood-derived biomaterials and has much denser, larger, and richer in growth factors fibrin matrix than platelet-rich plasma (PRP) and platelet-rich fibrin (PRF) [15]. It has been found that CGF is involved in the gingival regeneration by activation of AKT/Wnt and YAP signaling pathway [16] and osteogenesis following tooth extraction [17]. However, few clinical reports investigated the function of CGF on soft tissue closure.

Therefore, the objectives of this study was to compare the therapeutic effect of Bio-Gide^®^ collagen membrane and CGF membrane as sealing material in ARP.

## Materials and methods

### Patient selection

The study is a randomized RCT conducted in a manner consistent with the 1975 Declaration of Helsinki and its amendments since 2000. The study protocol was approved by the ethical committee of The Affiliated Stomatological Hospital of Xuzhou Medical University (Approval Number: 2021-002), and registered in Chinese Clinical Trial Registry (ChiCTR2100049442). Written consent forms were signed by all patients and the potential risks of the study were made known to all patients.

The inclusion criteria were as follows: > 15 years old; no systemic diseases, no active periodontal disease, and plan for a dental implant-supported restoration. The following criteria were used to exclude patients: an excessive smoker (> 5 cigarettes/day); periodontitis untreated or poor oral hygiene; previous history of irradiation of the head and neck area; pregnant; uncontrolled diabetes; current or past treatment with bisphosphonate; inability to complete the follow-up; at least half of the alveolar buccal bone plate remained after tooth extraction. The same specialist performed two surgical procedures on selected patients: (1) minimally invasive tooth extraction and ARP; (2) placement of implants after 6 months of ARP.

### CGF preparation

Nine mL venous blood was collected from patients and was stored in sterile vacuum tube (Greiner Bio-One, GmbH, Kremsmunster, Austria) without any anticoagulant. Then, the tube was immediately placed in centrifuge (Medifuge, Silfradentsrl, Italy) with fixed process: acceleration for 30 s, 2700 rpm for 2 min, 2400 rpm for 4 min, 2700 rpm for 4 min, 3000 rpm for 3 min, deceleration to a stop for 36 s. After this centrifugation process, the CGF was composed of three sections including an upper layer consisting of serum, light yellow gelatin in the middle which consisted of lots of growth factors at the junction with the lower layer, and a lower layer containing the red blood cells (RBCs). Solid CGF was extracted from each tube after centrifugation with sterile tweezers. The lower RBCs were cut away, the fibrin layer and the junction of the fibrin were then using gauze “squeezed” to form CGFs membranes for cover tooth extraction wound.

### Surgical procedures

Firstly, minimally invasive tooth extraction was performed and attention should be paid to protect the alveolar bone plate and surrounding soft tissues, rinsing the extraction socket with sterile saline. After examining the socket and debriding it, the inflammatory granulation tissues should be completely removed. Patients were randomly assigned to one of two groups. For the CGF group, the sockets were grafted with collagen-enriched deproteinized bovine bone mineral (Bio-Oss^®^ Collagen, Geistlich, Switzerland) and covered with CGFs membranes, stabilized with a suture. For the Bio-Gide group, the wound was covered with collagen membranes (Bio-Gide^®^, Geistlich, Switzerland) and the rest of the operations were the same as the CGF group.

**Fig. 1:**
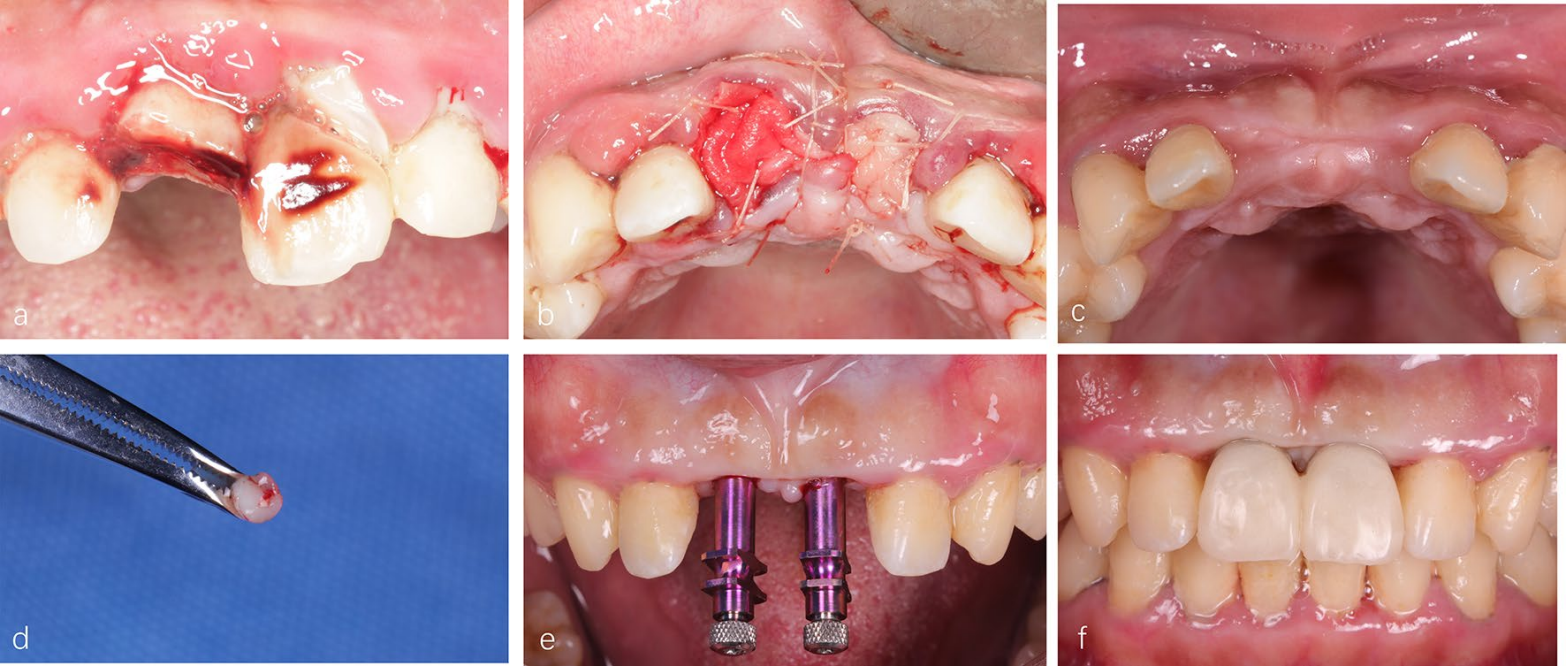
The process of ARP. **a** Clinical conditions at baseline; **b** 100 mg Bio-oss^®^ Collagen was placed in both extraction sockets, 11 surfaces were covered with Bio-Gide^®^ collagen membranes, 21 were covered with CGFs membranes; **c** clinical conditions at the 6-month follow-up; **d** the soft tissue sample from implant surgery; **e**, **f** the initial stability of the two implants during the operation was greater than 35 Ncm, the immediate postoperative repair was selected

Patients were instructed to take Roxithromycin and Ornidazole (North China Pharmaceutical, China) twice a day for 3 days and rinse with 0.2% chlorhexidine (Jiangsu Chenpai Bond Pharmaceutical , China). All patients were asked to follow up at 3,7,14 days and suture removal after 7 days. Six months after extraction, CBCT scans were performed and dental implants were placed using a minimally invasive technique. Meanwhile, the implant site was initially prepared with a soft tissue punch. A trephine (external diameter of 3 mm, internal diameter 2 mm) for harvesting a soft tissue sample during the implant surgery. If the initial stability of the implant was greater than 35 Ncm, the immediate repair will be considered (Fig. 1).

### Outcome measures

#### Soft tissue healing

Area measurement is one of the most commonly used methods for assessing wounds in clinical and research settings. According to literature reports the 3D Wound Reconstruction System is the most precise and accurate device currently available for assessing wound size [18].

**Fig. 2:**
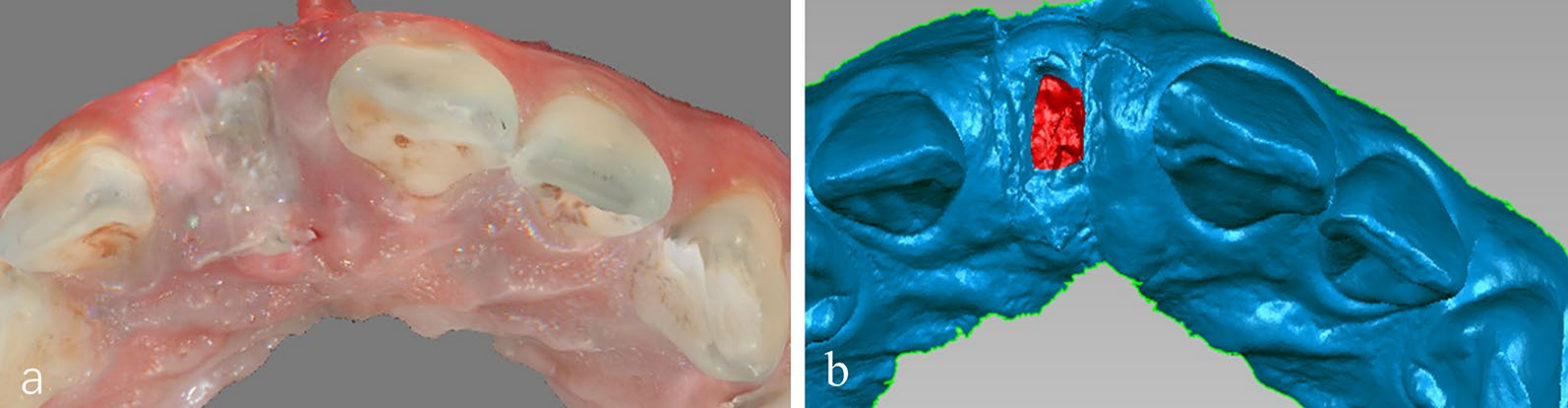
The representative pictures of intraoral scanner. **a** Wounds are recorded using oral scanning software; **b** the area identified for assessment of area changes is demarcated in red

Follow-up visits were performed on 3, 7, 14 days after ARP, and digital oral scanning equipment (CS 3600 Carestream) was used to scan the patient’s operating area and adjacent dentition (the adjacent teeth will be used as reference landmarks for subsequent model registration) to obtain STL files (Fig. 2a). Then import the file into Geomagic Studio 2014 to measure the wound area (Fig. 2b), calculate the healing rate according to the calculation formula of healing rates reviewed by Jessup [19] for the wound healing rate:

$$\left( {\left( {{\text{Area}}0 \, - {\text{Area}}t1} \right)/\left( {{\text{Area}}0} \right)} \right) \times 100\% .$$

### Keratinized gingiva width

**Fig. 3:**
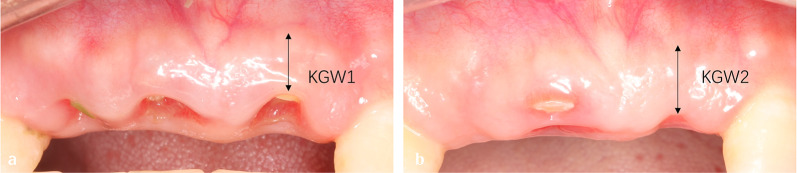
**a** Alveolar ridge preservation before surgery; **b** Alveolar ridge preservation 6 months

The distance from the buccal central gingival margin to the mucogingival symphysis was measured preoperatively recorded as KGW1 (Fig. 3a), and the distance from the buccal central alveolar ridge to the mucogingival symphysis was measured 6 months after operation, it was recorded as KGW2 (Fig. 3b). Variation in keratinized gingiva width = KGW2-KGW1 [20].

### CBCT analysis

**Fig. 4:**
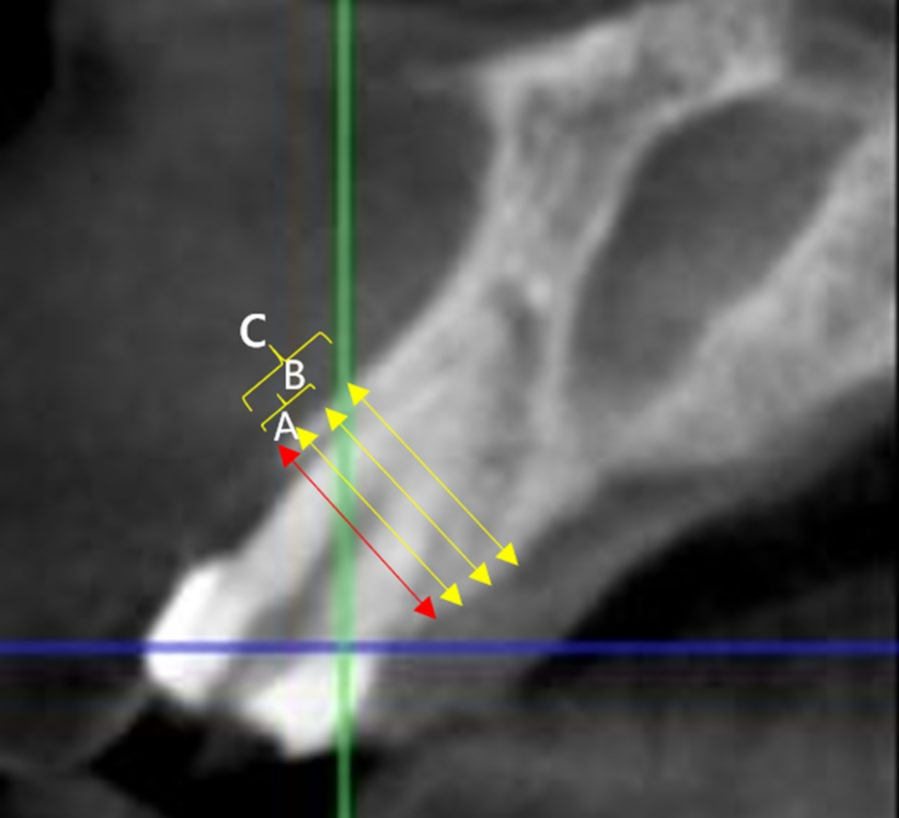
Volumetric changes. horizontal measurements. The red line is the baseline, **A** line is 1 mm below the reference line, **B** line is 3 mm below the baseline line, and **C** line is 5 mm below the baseline line

In both groups, CBCT scans were conducted prior to the extraction and six months after the ARP procedure. Three measures were recorded for all preserved sites, before and after treatment. There were three horizontal ridge widths measured at three different levels located 1, 3 and 5 mm below the most coronal aspect of the bone crest, respectively (Fig. 4). Each level’s bone loss was expressed as a linear difference between pre- and post-regeneration measurements.

### Histologic analyses

Nascent soft tissue samples were adequately fixed in 4% paraformaldehyde. Routine dehydration-embedded sections, immunohistochemical staining with SP immunohistochemistry kit to observe the expression of the vascular marker CD31 in the gum tissue. Blood vessel density (MVD) count Light microscopy (Nikon Eclipse E100, Japan) to observe the expression of CD31 in each group of gum tissue sections.

### Statistical analysis

The data were analyzed statistically with SPSS 22.0. The differences of means at the patient level for continuous outcomes (horizontal volumetric changes, soft tissue healing rate, change of keratinized gingiva width and micro-vessels density) between groups were compared by independent sample *t*-tests.

In order to estimate whether the data were normally distributed, Shapiro–Wilk was used, and homogeneity was assessed using the homogeneity of variance test. If the data follow a normal distribution with the same variance then the independent samples *t*-test is used. All data were averaged by the same person after three measurements. A significance level of α = 0.05 was used for all analyses.

## Results

### Study population

A total of 22 patients (11 men and 11 women) with 24 sites. All patients met the requirements for implant surgery after 6 months of ARP. The patients’ average age was 30.46 ± 10.58 years (range 19–61 years). Patients experienced no complications during the surgery, and all had uneventful healing. The tooth extraction site information is described in detail in Table 1.

**Table 1:** Patient and tooth extraction site characteristics

Parameter	Group	
	CGF	Bio-Gide
Patients	11	11
Gender		
Male	9	2
Female	8	3
Age, y		
Mean ± SD	30 ± 10.39	32.2 ± 12.38
Range	19–51	19–61
Implants in incisor position	11	10
Implants in premolar position	1	2

### Soft tissue healing

**Fig. 5:**
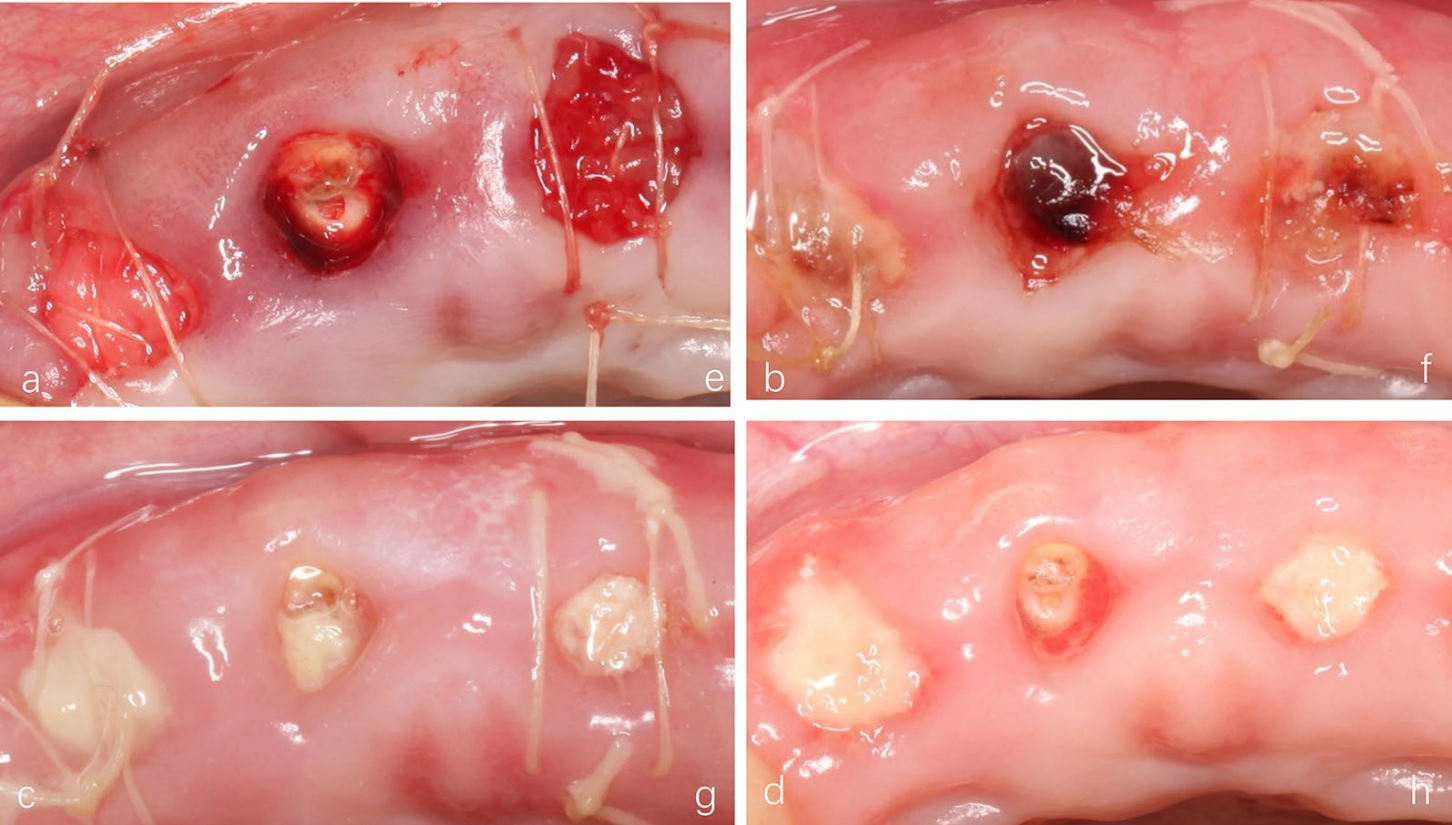
Soft tissue healing at different times. **a**–**d** Images at 3, 7 and 14 days postoperation in Bio-Gide group. **e**, **f** Images at 3, 7 and 14 days postoperation in CGF group

**Fig. 6:**
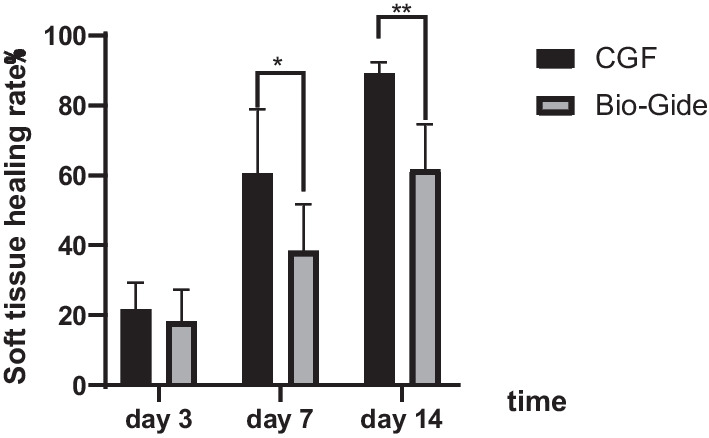
Comparison of changes in wound healing rate at 3, 7 and 14 days after ARP (*P < 0.05**, *P* < 0.001)

All patients were followed up at the required time, and there were no cases of infection. And the soft tissue healing in the CGF group was significantly better than that in the Bio-Gide group (Fig. 5). The soft tissue healing rate of the CGF group was 21.71% ± 7.68% at 3 days, and the soft tissue healing rate of the Bio-Gide group was 18.19% ± 9.11%. There was no statistical difference between the two groups (*P* = 0.317). However, at 7 days after surgery, the soft tissue healing rate of the CGF group was 60.51% ± 18.41%, and the healing rate of the Bio-Gide group was only 38.38% ± 13.37%, with a statistically significant difference between the two groups (*P* = 0.003). At 14 days after the operation, the soft tissue healing rate in the CGF group was 89.1% ± 3.21%, and the healing rate in the Bio-Gide group was 61.73% ± 12.92%, and there was significant difference between the two groups (*P* < 0.001) (Fig. 6).

### Change of keratinized gingiva width

**Fig. 7:**
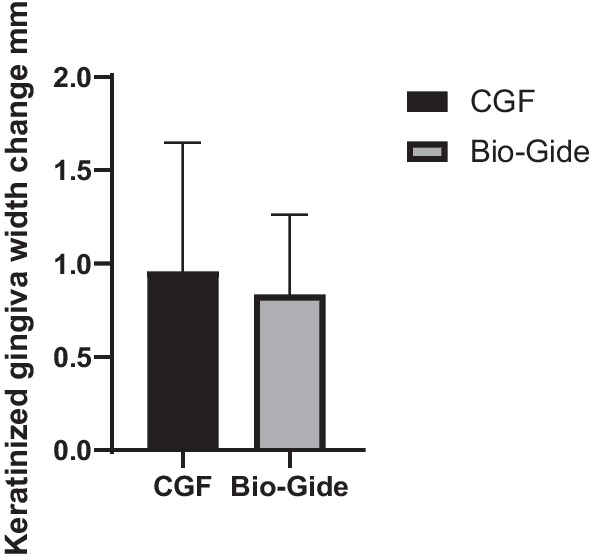
Change of keratinized gingiva width in CGF and Bio-Gide group

Six months after alveolar ridge preservation of healing, the wounds of 22 patients were completely healed. The increase in keratinized gingiva width in the CGF group was 0.985 ± 0.6895. The change in the Bio-Gide group was 0.833 ± 0.4292. There was no statistical difference between the two groups (*P* = 0.599) (Fig. 7).

### CBCT analysis

Alveolar ridge dimensions in all patients at 6 months were considered acceptable for implantation by minimally invasive surgery and does not require additional guided bone regeneration during surgery, which defining 100% clinical efficacy of ARP surgery. The labial and palatal plates were attached to a reference baseline prior to extraction, and the reference baseline was used as a standard for subsequent measurements.

**Fig. 8:**
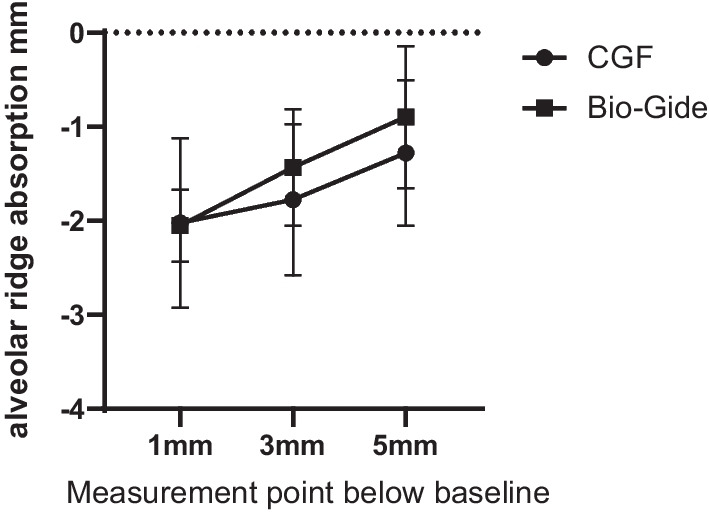
Changes of alveolar ridge width 6 months after ARP in CGF group and Bio-Gide group

After 6 months, the width change at 1 mm below the baseline was − 2.02 mm ± 0.9 mm in the CGF group and − 2.1 ± 0.38 mm in the Bio-Gide group. There was no statistical difference between the two groups (*P* = 0.917). The absorption of 3 mm CGF under the alveolar ridge was − 1.77 ± 0.8 mm, and the absorption of the Bio-Gide group was − 1.43 ± 0.62 mm. There was no statistical difference between the two groups (*P* = 0.327). The change in the width of 5 mm under the alveolar ridge in the CGF group was − 1.27 ± 0.76 mm, and the change in the Bio-Gide group was − 0.9 ± 0.76 mm, no statistical difference between the two groups (*P* = 0.327) (Fig. 8).

### Histological analyses

**Fig. 9:**
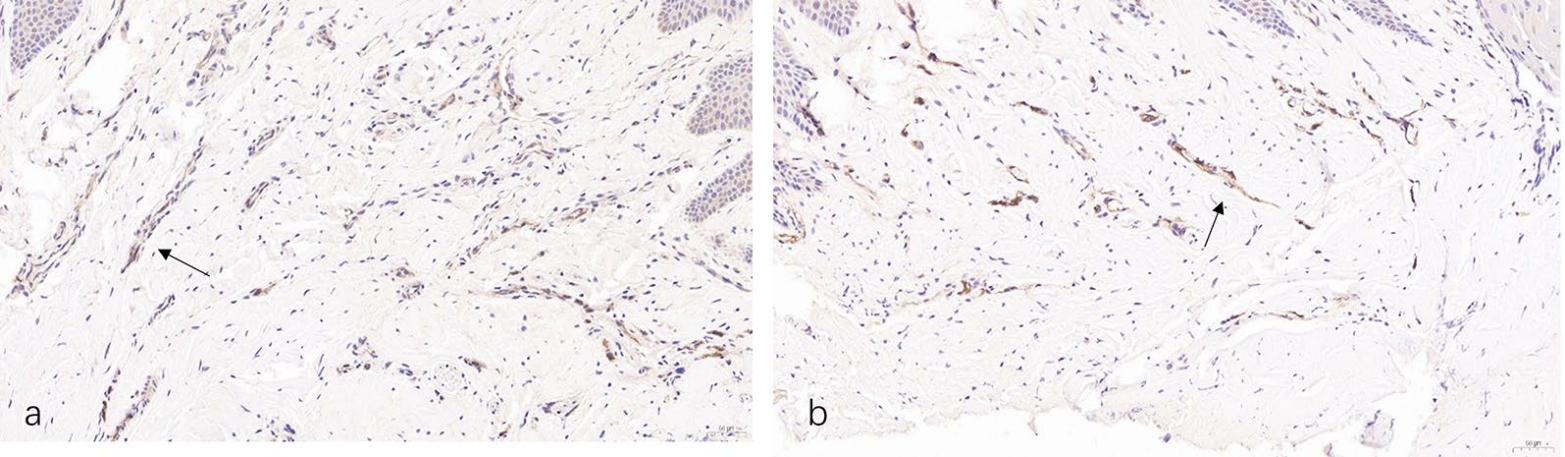
Immunohistochemical staining CD31. **a** CGF group. **b** Bio-Gide group

**Fig. 10:**
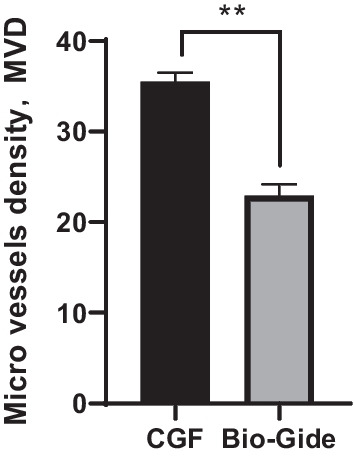
Micro-vessels density (MVD) in CGF and Bio-Gide group

Immunohistochemical staining of gingival soft tissue specimens of CD31 expression positive cells is pale yellow or brownish-yellow (shown by the black arrow) (Fig. 9). The more positive signals, the more neovascularization of the gum soft tissue, the better the healing of the soft tissue. All vascular endothelial cells in both groups of specimens were positive for CD31 expression. Meanwhile, the density of new blood vessels was counted by CD31 immunohistochemical staining in the soft tissue obtained during the operation. The average number of new blood vessels in the CGF group was 35.32 ± 3.47, which was significantly higher than that in the Bio-Gide group which was 22.93 ± 4.42 (*P* < 0.001) (Fig. 10).

## Discussion

This study evaluated the healing rate of soft tissue and the change of alveolar bone which using different sealing materials in ARP. The literature shows that alveolar preservation techniques are more effective than natural healing [21, 22]. In order to compare only one variable, this study did not include natural healing group, and Bio-Oss Collagen was used in all patients. Results indicated that a complete preservation of the alveolar crest with ARP technique is unlikely and there was no significant differences between CGF groups and Bio-Gide groups concerning the alveolar height changes. Previous studies have reported the similar result [23].

Free gingiva, resorbable collagen membranes and non-resorbable collagen membranes are the most commonly used sealing materials in ARP. Previous studies have shown that there was no statistical difference among these three materials in terms of the effectiveness of extraction site preservation [24], but all of them have shortcomings: the use of free gingival closure requires the creation of a second operative area and the free tissue was prone to necrosis [25]; the use of non-absorbable collagen membranes requires secondary surgical removal and the use of absorbable collagen membranes was more expensive.

As an autologous blood extract, CGF has the advantages of low cost, convenient collection, and mild postoperative response. In recent years, CGFs membranes were used in guided bone regeneration to promote soft tissue healing [26]. In addition, the high concentration of anti-infection factors in CGF reduced the likelihood of postoperative infections [27]. It was found in several studies that researchers filled CGF in combination with bone grafting material in extraction sockets or put CGF alone for ARP. For example, Lin et al. [28] used CGF gels mixed with DBBM then filled this mixture in the socket and covered with CGFs membranes in the test group, while filled DBMM alone and covered with Bio-Gide® collagen membranes in control group. Mixing CGF with DBBM makes it impossible to objectively evaluate the effect of CGF on wound closure. It can be seen that there is no research on the healing effect of CGFs membranes only on soft tissue in alveolar ridge preservation.

In this study, CGFs membranes were used to seal the socket and the wound healing rate was used to evaluate the effect of soft tissue closure. Many investigations on soft tissue healing have been performed by a modified version of the Masse healing index (HI) [29]. However, this method has the disadvantage of being highly subjective and not reflecting the rate of wound healing. Therefore, we used an intraoral scanner to obtain soft tissue information from patients at 3, 7, and 14 days in equal proportions, counted the wound area by Geomagic 2014 software and evaluated the wound healing effect by the soft tissue healing rate. These quantified data making the conclusions more accurate and reliable. The width of keratinized tissue may be important in maintaining periodontal health and preventing soft tissue recession [30], Chung et al. [31] reported that lack of keratinized mucosa predisposes to peri-implantitis. It can be seen that the width of the keratinized gingiva also has an important influence on implant surgery, so this study also measured the width of the keratinized gingiva. The width of the keratinized gingiva was increased in both groups of patients 6 months after alveolar ridge preservation. The amount of increase was not statistically different (*P* > 0.05), which proved that CGF and Bio-Gide membrane had the same effect on the healing of keratinized gingiva. Zhang et al. [32] came to the same conclusion.

Immunohistochemistry was used to observe neurovascular regeneration in soft tissue between the two groups 6 months after surgery to determine whether CGF could promote soft tissue healing. It can be seen that sites using CGFs membranes significantly increased the number of blood vessels positive for the vascular endothelial cell marker CD31. CGFs seem to have the potential to accelerate soft tissue healing earlier than Bio-Gide for the reason that most collagen membranes are known to release glutaraldehyde during healing, which probably led to cell death and dysfunction [33].

From the data obtained from CBCT, the amount of bone resorption with CGFs membranes was not statistically different from that with Bio-Gide after 6 months of ARP. According to Cardaropoli et al. [34] Bio-Oss^®^ could be used to slow alveolar ridge resorption and stimulate new bone formation. Furthermore, the combination of DBBM with Bio-Gide^®^ collagen membranes may significantly reduce the vertical and horizontal resorption of alveolar bone. This was similar to our findings: the use of CGF and Bio-Gide can play a good sealing effect and preserve the bone mass of alveolar ridge and provide favorable conditions for later implant surgery. This study also compared the ability of alveolar bone to resorb horizontally and both groups achieved similar effects. Silvio et al.[7] used porcine collagen matrix + DBBM, the horizontal bone resorption at 1, 3, and 5 mm at baseline was 0.67 ± 0.31 mm, 0.91 ± 0.38 mm, and 0.31 ± 0.18 mm after 5 months. In a systematic review that included 32 randomized controlled clinical trials, Jambhekar et al.[35] evaluated the ARP effect of DBBM filling analyzed the ARP effect of DBBM filling, which showed that the reduction in the horizontal width of the alveolar bone was 1.30 mm. These were similar to our results, but there was a certain difference in the amount of numerical change, which we think was due to the measurement method and the error caused by different CT imaging equipment.

Although CGF has many advantages, there are also shortcomings, such as easy to fall off and individual patient differences, and the next step is to increase the sample size to better evaluate the effect. We also need to develop a standardized procedure for CGF extraction and to improve the procedure for CGFs membranes fixation.

## Conclusion

The application of CGFs membranes in ARP is a simple and cost-effective method, and has faster soft tissue healing speed and similar bone formation compared with Bio-Gide^®^ membranes. Therefore, CGF could be recommended to patients with alveolar ridge preservation as a better choice considering economical and safe factors. However, our results need to be confirmed with larger sample sizes and longer follow-ups.

## Data Availability

Not applicable.
